# Integrating single-cell and bulk RNA sequencing data reveals RGS4 as a functional driver in a proliferative subgroup of SF-1 lineage PitNETs

**DOI:** 10.3389/fcell.2026.1815682

**Published:** 2026-04-22

**Authors:** Changxi Han, Qiuyue Fang, Bo Zhang, Ying Yuan, Can Xu, Jin Yang, Yangyang Wang, Mingjun Gao, Huan Xiong, Ruxiang Xu

**Affiliations:** 1 Department of Neurosurgery, Sichuan Provincial People’s Hospital, University of Electronic Science and Technology of China, Chengdu, China; 2 Department of Neurosurgery, Center of Pituitary Tumor, Ruijin Hospital, Shanghai Jiao Tong University School of Medicine, Shanghai, China

**Keywords:** p53, PitNETs, proliferation, RGS4, SF-1

## Abstract

**Introduction:**

The molecular determinants underlying the heterogeneity and proliferative behavior of Steroidogenic Factor-1 (SF-1) lineage Pituitary Neuroendocrine Tumors (PitNETs) remain insufficiently defined.

**Methods:**

We performed an integrated analysis of single-cell RNA sequencing (scRNA-seq) and bulk transcriptomic datasets from SF-1 lineage PitNETs to identify molecular subgroups and their defining biomarkers. The functional significance of the top candidate gene, Regulator of G-protein Signaling 4 (RGS4), was examined using gain- and loss-of-function approaches and pharmacological inhibition in pituitary adenoma cell lines. Mechanistic studies were conducted using protein interaction and stability assays.

**Results:**

We identified a previously unrecognized SF-1 lineage subgroup characterized by markedly elevated *RGS4* expression, which correlated with larger tumor size and higher MIB-1 proliferation index. Functional experiments demonstrated that *Rgs4* enhances tumor cell proliferation and inhibits apoptosis. Mechanistically, RGS4 promotes p53 ubiquitination, resulting in its destabilization and proteasomal degradation.

**Conclusion:**

This study defines a new SF-1 lineage PitNETs subgroup characterized by high-*RGS4* expression. RGS4 promotes tumor proliferation by destabilizing p53, highlighting the RGS4-p53 axis as a promising therapeutic target.

## 1 Introduction

Pituitary neuroendocrine tumors (PitNETs), commonly known as pituitary adenomas, account for approximately 10%–15% of all intracranial tumors and represent a major source of morbidity due to their compressive effects and associated endocrine dysfunction (Asa et al., 2022; Neou et al., 2020). Among these, clinically non-functioning PitNETs (NF-PitNETs), which do not manifest hormone hypersecretion syndromes, are typically diagnosed at a larger tumor size owing to their silent clinical course (Wang et al., 2022). A substantial subset (30%–50%) of NF-PitNETs arises from the gonadotroph lineage, defined by expression of the transcription factor Steroidogenic Factor-1 (SF-1, encoded by NR5A1) (Nishioka et al., 2015; Campbell et al., 2024; Meinsohn et al., 2019). Although often categorized as “silent,” SF-1 lineage PitNETs impart significant clinical burden, frequently leading to visual field impairment and hypopituitarism due to mass effects. Although transsphenoidal surgery remains the primary treatment, complete resection is frequently hindered by cavernous sinus invasion, leading to tumor residuals and high recurrence rates. Postoperative radiotherapy is often employed for residual or recurrent tumors, but it carries risks of hypopituitarism, optic neuropathy, and secondary malignancies. These unresolved challenges, including surgical limitations, radiotherapy-related morbidity, and the absence of targeted drug therapies, underscore the urgent need to investigate the molecular mechanisms driving tumor proliferation, which may reveal novel therapeutic targets for this clinically recalcitrant disease (Maldar et al., 2022; Agu et al., 2015; Bernat et al., 2021).

Recent advances in transcriptomic technologies have significantly expanded our understanding of the molecular complexity of PitNETs. Comprehensive genomic and transcriptomic investigations have delineated distinct molecular subgroups across different tumor lineages, laying the groundwork for refined molecular classification and risk stratification (Arneth, 2019; Toth, 2023; Cui et al., 2021). Recent work by Su et al. identifies an aggressive tumor cluster marked by elevated p53-mediated proliferation and a higher Trouillas classification in TPIT-lineage PitNETs (Su et al., 2025). Lyu et al. build up a cellular landscape of PIT1-lineage PitNETs TME and highlights anti-tumour function of IFN-γ mediated TAF remodelling (Lyu et al., 2023). Although pioneering single-cell atlases have mapped the broader cellular heterogeneity of PitNETs (Su et al., 2025; Lin et al., 2024), the intratumoral compositional diversity within the SF-1 lineage, particularly in terms of the relative proportions and states of tumor cells, immune infiltrates, and stromal components, remains poorly defined. This knowledge gap hampers the discovery of context-specific cellular states and cell-intrinsic molecular drivers that govern tumor aggressiveness and proliferative behavior. To address this unmet need, our study employs an integrated single-cell and bulk RNA sequencing data to systematically characterize the cellular architecture and functional heterogeneity of SF-1 lineage PitNETs, to uncover novel and therapeutically actionable vulnerabilities unique to this clinically challenging tumor subtype.

The molecular determinants underlying the progression of SF-1 lineage PitNETs remain incompletely understood. The tumor suppressor p53, a master regulator of cell-cycle arrest and apoptosis, is mutated in a subset of highly aggressive PitNETs but is more commonly retained in its wild-type form (Lee et al., 2021; Raverot et al., 2021). In many cancers, p53 function is compromised either by TP53 mutations or by dysregulation of its regulatory network, including overexpression of negative regulators such as MDM2 and MDM4 (Koo et al., 2022; Jain, 2020). However, TP53 mutations are relatively infrequent compared to other malignancies, suggesting alternative mechanisms of p53 pathway inactivation in PitNETs (Yao et al., 2023; Wang et al., 2020). In such cases, post-translational mechanisms—particularly ubiquitin-mediated degradation—play a central role in controlling p53 protein stability and functional activity across many cancer types (Wang et al., 2023). However, whether p53 plays an essential role in PitNETs biological behavior and how it is regulated is poorly understood.

Accumulating evidence highlights the involvement of G-protein signaling proteins, especially RGS4, in modulating cellular proliferation, survival, and therapeutic responses in several malignancies, including glioblastoma and osteosarcoma (Tan et al., 2025; Ben et al., 2025; Guda et al., 2020). Despite this emerging relevance, the role of RGS4 in pituitary tumorigenesis remains virtually unexplored. Although RGS4 is well characterized for its canonical GTPase-activating function that attenuates Gαi/o and Gαq signaling (Luessen et al., 2017), its potential interactions with key tumor-suppressive pathways, notably the p53 axis, have not yet been investigated in the context of PitNETs.

In this study, we integrated single-cell RNA sequencing (scRNA-seq) and bulk transcriptomic profiling to characterize cellular heterogeneity within SF-1 lineage PitNETs, focusing on the subgroup with high *RGS4* expression. We further demonstrated that RGS4 drives tumor proliferation and suppresses apoptosis by promoting ubiquitin-mediated degradation of p53. These findings advance the molecular subtyping of SF-1 PitNETs and nominate the RGS4-p53 axis as a potential therapeutic target for this challenging tumor subtype.

## 2 Materials and methods

### 2.1 Data acquisition

The raw scRNA-seq and bulk RNA-seq datasets used in this study were obtained from the Genome Sequence Archive (GSA) at the National Genomics Data Center, China National Center for Bioinformation/Beijing Institute of Genomics, Chinese Academy of Sciences, under the accession number GSA-Human: HRA005096. The dataset was originally generated and published by Lin et al. (2024).

### 2.2 Single-cell quality control

We imported the gene expression matrices into Seurat (R package) for quality control and downstream analysis. Cells expressing fewer than 200 genes, more than 5,000 genes, possessing >50,000 unique molecular counts, or exhibiting >30% mitochondrial RNA content were excluded. After applying these filters, 26,376 high-quality cells were retained for further analysis. Potential doublets were identified and removed using DoubletFinder with default parameters. To minimize ambient RNA contamination, we applied decontX, which effectively eliminated background RNA signals.

### 2.3 Batch effect adjustment

We identified the top 2,000 highly variable genes and used them to perform principal component analysis (PCA). To correct for batch effects across samples, we applied the Harmony algorithm (R package), setting the max. iter. harmony parameter to 5. The top 15 Harmony-derived coordinates were then utilized for downstream clustering and dimensionality reduction analyses.

### 2.4 Clustering and dimensionality reduction

Graph-based unsupervised clustering was performed using a resolution of 0.25 for whole-cell clustering and 0.07 for SF1 tumor cell re-clustering. Uniform Manifold Approximation and Projection (UMAP) was applied for initial visualization of the cellular landscape.

### 2.5 Annotation of major cell populations

After the clustering, eight distinct cell clusters were identified. Cluster-specific marker genes were determined using the FindAllMarkers function, applying the following thresholds: log2 fold change >0.25, min.pct >0.25, and adjusted P-value <0.05. Cell-type annotation was subsequently performed based on the expression patterns of canonical marker genes.

### 2.6 Differentially expressed genes calculation

Differentially expressed genes were identified using the limma package, comparing expression profiles across distinct cell types. The log fold change (logFC) threshold was set at 1, and the adjusted-p-value threshold was set at 0.05.

### 2.7 Functional enrichment analysis

Genes were ranked based on their log_2_ fold change (log_2_FC) values, and functional annotation was performed using the Gene Ontology Biological Process database. Enrichment significance was determined using thresholds of p-value <0.2 and q-value <0.2. The enrichplot R package was used to visualize the enrichment results.”

### 2.8 Transcription factor (TF) enrichment analysis

Transcription factor activity underlying transcriptomic alterations was inferred using the DoRothEA v2 framework. Regulons with confidence levels A-C were included in the analysis, and significant enrichment was defined by a p-value <0.05 and a normalized enrichment score (NES) ≥ |2|

### 2.9 Non-negative matrix factorization (NMF) algorithm

Non-negative matrix factorization (NMF) was applied to cluster the Gonadotroph samples. The analysis was performed using the ‘brunet’ algorithm. nRuns was set to 100. The number of clusters (k) was evaluated across a range of 2–10, and clustering quality was assessed using the consensus matrix, implemented in the NMF R package, with a minimum subclass size of 10 samples. Based on the cophenetic curve, the optimal number of clusters was determined to be three.

### 2.10 Deconvolution of cellular composition

The cellular composition in the bulk tumor samples was inferred using CIBERSORT in R. A custom signature matrix was generated from our single-cell RNA-seq dataset by aggregating marker gene expression profiles for the G1, G2, and G3 subgroups. The package was executed in B-mode with 10 permutations to estimate the relative proportions of each cellular state in the bulk RNA-seq samples. These inferred fractions were subsequently used for correlation and clustering analyses.”

### 2.11 Calculation of p53 pathway activity scores

Pathway activity of the p53 signaling cascade in the bulk RNA-seq dataset was assessed using single-sample Gene Set Enrichment Analysis (ssGSEA) implemented in the GSVA R package. The HALLMARK ‘p53 signaling pathway’ gene set was retrieved from the Molecular Signatures Database (MSigDB). ssGSEA was performed on the normalized expression matrix to compute enrichment scores for each sample, reflecting the relative activation level of the p53 pathway.

### 2.12 RNA sequencing

Total RNA from AtT20 cells was isolated using the AllPrep® DNA/RNA Mini Kit (QIAGEN, Hilden, Germany). RNA-seq libraries were prepared with the NEBNext® Ultra™ Directional RNA Library Prep Kit for Illumina® (NEB, Ipswich, MA, United States) and sequenced on an Illumina platform to generate 150-bp paired-end reads. Clean reads were aligned to the *Rattus norvegicus* reference genome (Rnor_6.0) using HISAT2 (v2.0.5). Gene expression levels were quantified as fragments per kilobase per million mapped reads (FPKM) and transcripts per million (TPM).

### 2.13 Cell culture and reagents

The AtT20, GH3, and MMQ cell lines were obtained from the American Type Culture Collection (ATCC, Manassas, VA, United States). Cells were maintained in Ham’s F-12K medium (L450KJ, BasalMedia) supplemented with 2.5% FBS (S615JY, BasalMedia), 15% horse serum (26050088, ThermoFisher), and 1% penicillin/streptomycin (C100C5, NCM Biotech). All cultures were incubated at 37 °C in a humidified atmosphere with 5% CO_2_. The following reagents were used in subsequent experiments: cycloheximide (CHX, HY-12320, MedChemExpress) and CCG-50014 (HY-13509, MedChemExpress).

### 2.14 Tissue dissociation and primary tumor cell extraction

PitNET tissues were transported on ice in DMEM (Gibco, 11875–093) without FBS containing 1 mM protease inhibitor (Solarbio, P6730). After washing three times with PBS, tissues were minced on ice, cut into pieces and digested in serum-free DMEM with an enzyme cocktail consisting of 1 U/mL DNase I (NEB, M0303S), 1 mg/mL Type VIII Collagenase (Sigma-Aldrich, C2139), 1 mg/mL Trypsin Inhibitor (Sigma-Aldrich, T6522) and 2 mg/mL Dispase II (Sigma-Aldrich, 4942078001). Digestion was performed at 37 °C, followed by shaking at 50 rpm for approximately 40 min. The suspension was filtered through a 40-μm strainer (Falcon, 352340), and red blood cells were removed using RBC lysis buffer (beyontime, C3702). Cells were then washed with PBS using a stepwise descending centrifugation. Viability was assessed by Trypan blue staining (Invitrogen, T10282), and cells were subsequently cultured in DMEM supplemented with 10% FBS and 1% antibiotic mixture.

### 2.15 CellTiter-Glo luminescence assay

Cell viability was evaluated using the CellTiter-Glo Luminescent Assay (G9241, Promega). A total of 500 cells per well were seeded into 96-well plates in complete medium. CellTiter-Glo reagent was added at a 1:1 ratio to each well, and luminescence was recorded following a 10-min incubation at room temperature using a microplate reader. Data from at least three independent experiments were normalized to vehicle-treated controls and analyzed in GraphPad Prism using an unpaired Student’s t-test or two-way ANOVA.

### 2.16 Cell apoptosis assay

Apoptosis was assessed by flow cytometry using the Annexin V-FITC Apoptosis Detection Kit (AD10, Dojindo). Tumor cells were collected and washed twice with PBS, followed by staining with Annexin V-FITC and propidium iodide (PI) according to the manufacturer’s protocol. Stained cells were analyzed on a BD FACSCalibur flow cytometer, and data were processed using FlowJo software. The proportion of apoptotic cells was subsequently quantified.

### 2.17 Real-time RT-PCR

Total RNA was extracted from cells samples using TRIzol reagent (Accurate Biology, AG21102) following PBS washing. cDNA was synthesized from 1 µg of RNA using the Evo M-MLV RT Mix Kit with gDNA Clean (Accurate Biology, AG11728) according to the manufacturer’s instructions. Quantitative PCR was performed on an ABI 7500 Real-Time PCR System (Thermo Fisher) using SYBR Green Master Mix (Accurate Biology, AG11718). β-actin served as the internal control, and relative gene expression levels were calculated using the 2^−ΔΔCt^ method. Primer sequences are listed in Additional File 2, and all samples were analyzed in triplicate.

### 2.18 Western blotting

Total protein was extracted from cells or tissues using RIPA lysis buffer (Beyotime Biotechnology, P0013C) supplemented with protease and phosphatase inhibitors (NCM Biotech, P002). Protein concentrations were quantified using a bicinchoninic acid (BCA) assay kit (Yoche, YSD-500T). Equal amounts of protein were denatured, resolved on SDS–PAGE gels, and transferred onto polyvinylidene difluoride membranes. After blocking, membranes were incubated overnight at 4 °C with primary antibodies (listed in Additional File 1), washed, and subsequently treated with horseradish peroxidase-conjugated secondary antibodies for 1 h at room temperature. Protein bands were detected using an enhanced chemiluminescence kit (Servicebio, G2020-25ML) and imaged accordingly. All Western blot data presented are representative of three independent biological replicates.

### 2.19 Immunoprecipitation (IP) assay for ubiquitination analysis

IP assays were conducted to evaluate the impact of RGS4 on p53 ubiquitination under two experimental conditions. In the first condition, 293T cells were co-transfected with plasmids encoding HA-tagged ubiquitin (HA-Ub), 3×FLAG-tagged p53 (3×FLAG-p53), and an *RGS4* overexpression construct. In the second condition, cells were co-transfected with HA-Ub and p53-3×FLAG plasmids and subsequently treated with the RGS4 inhibitor CCG-50014 at 24 h post-transfection. For both conditions, cells were exposed to 10 µM MG132 for 6 h before harvesting to prevent proteasomal degradation. Cells were lysed in RIPA buffer supplemented with protease inhibitors and 10 mM N-ethylmaleimide, and whole-cell lysates were incubated overnight at 4 °C with protein A sepharose beads pre-bound to anti-FLAG antibody. Immunocomplexes were washed thoroughly, eluted, denatured, and resolved by SDS-PAGE, followed by Western blotting with anti-HA and anti-FLAG antibodies to assess ubiquitination levels.

### 2.20 Stable cell lines

Stable cell lines were established via lentiviral transduction. Pituitary adenoma cell lines were infected with lentiviruses encoding Rgs4, sh*Rgs4*, or corresponding control constructs. After 48 h, transduced cells were subjected to puromycin selection for 7 days to generate stable polyclonal populations. Successful gene overexpression or knockdown was confirmed by RT-qPCR and Western blotting.

### 2.21 Subcutaneous xenograft

Female BALB/c nude mice (4 weeks old) were used for the xenograft experiments. AtT20, GH3, and MMQ cells were collected, washed with PBS, and resuspended in PBS at a concentration of approximately 2 × 10^6^ cells per 100 µL. Cell suspensions were injected subcutaneously at appropriate anatomical sites in each mouse. Once tumors reached a volume of approximately 100–200 mm^3^, length and width of tumors was measured every day. Tumor size was measured using a digital caliper, and volumes were calculated using the formula: length × width^2^ × 0.5.

### 2.22 Immunohistochemistry staining

Paraffin-embedded tissue sections were deparaffinized and rehydrated through a graded ethanol series. Following deparaffinization and rehydration, antigen retrieval was performed using an antigen unmasking solution. Endogenous peroxidase activity was blocked with H_2_O_2_, and non specific binding sites were blocked with normal goat serum. Sections were incubated with primary antibodies overnight at 4 °C, followed by detection using Dako REAL EnVision HRP (rabbit/mouse) and Dako REAL DAB + Chromogen.

### 2.23 Statistical analysis

All bioinformatics analyses were conducted using R version 4.2.1. P-values <0.05 were considered statistically significant and were adjusted for multiple testing using the Benjamini–Hochberg (BH) method. Experimental data were expressed as mean ± SEM and analyzed using GraphPad Prism. Comparisons between experimental and control groups were performed using unpaired t-tests with a 95% confidence interval. Statistical significance was denoted in figures as 1 asterisk (*p* < 0.05), 2 asterisks (*p* < 0.01), or 3 asterisks (*p* < 0.001).

## 3 Results

### 3.1 Single-cell transcriptomic profiling reveals the cellular landscape of SF-1 lineage PitNETs

**FIGURE 1:**
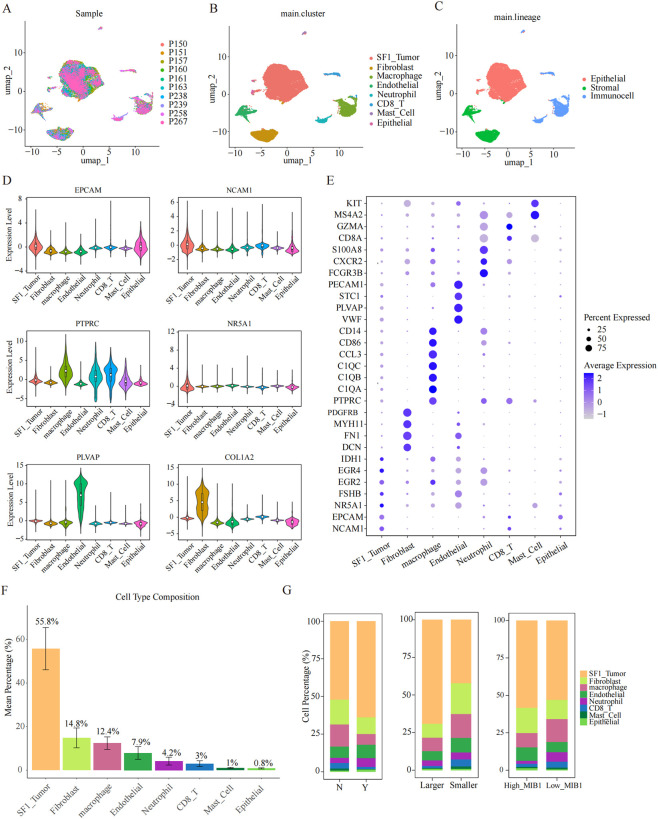
Single-cell analysis reveals the transcriptomic landscape in SF-1 lineage PitNETs. **(A)** UMAP visualization of the integrated dataset, color-coded by the 10 SF-1 lineage PitNETs samples. **(B)** Cell-type annotation identifying eight major cellular subpopulations within the tumor microenvironment. **(C)** UMAP highlighting the three primary lineages represented across the dataset. **(D)** Violin plots showing the expression patterns of canonical marker genes across the eight defined cell types. **(E)** Dot plot illustrating the specificity and expression intensity of representative marker genes associated with each of the eight clusters. **(F)** Bar chart depicting the relative abundance and proportional distribution of the eight cell populations in all samples. **(G)** Bar chart comparing the distribution of cell types between two tumor groups stratified by distinct biological characteristics.

To comprehensively delineate the tumor microenvironment (TME) of SF-1 lineage PitNETs, we performed a re-analysis of 10 SF-1 PitNETs samples from a previously published single-cell RNA-sequencing dataset (GSA-Human: HRA005096; Lin, S. et al.) (Lin et al., 2024). Unsupervised clustering of 26,376 high-quality cells identified eight major cellular populations (Figures 1A,B). Then, using well-established lineage-specific marker genes, these clusters were annotated as SF-1^+^ tumor cells, fibroblasts, macrophages, endothelial cells, neutrophils, CD8^+^ T cells, mast cells, and a minor epithelial cell subset. These populations were further grouped into three principal lineages within the PitNET TME: (i) Epithelial lineage, defined by EPCAM expression and comprising SF-1^+^ tumor cells and non-tumoral epithelial cells; (ii) Stromal lineage, characterized by COL1A2 and PLVAP expression and including fibroblasts and endothelial cells; and (iii) Immune lineage, marked by PTPRC expression and containing macrophages, neutrophils, CD8^+^ T cells, and mast cells (Figure 1C). Additional canonical markers were used to refine and validate cell-type annotations (Figures 1D,E). Among the epithelial lineage, SF-1^+^ tumor cells were specifically identified by co-expression of NR5A1, EPCAM, and NCAM1, whereas EPCAM^+^/NR5A1^-^/NCAM1^-^ clusters were classified as non-tumoral epithelial cells, consistent with the annotation reported by Lin et al. (Lin et al., 2024). Cellular composition analysis demonstrated that SF-1^+^ tumor cells represented the predominant population, accounting for an average of 55.8% of the total cell population (Figure 1F). Stromal components constituted 22.7% of cells, while immune populations accounted for 20.6%. Comparative assessments of tumors stratified by clinicopathological characteristics revealed marked differences in TME architecture (Figure 1G). Tumors with more aggressive biological features such as larger size, evidence of invasion, or elevated MIB-1 index showed a significantly higher proportion of SF-1^+^ tumor cells accompanied by a relative reduction in specific stromal and immune subsets.

**FIGURE 2:**
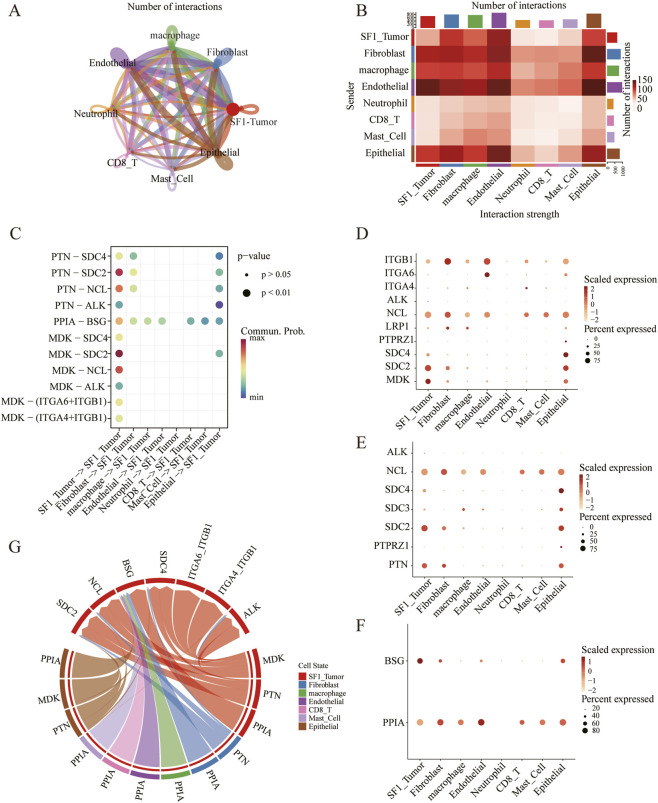
Intercellular communication networks within the SF-1 lineage PitNETs tumor microenvironment. **(A)** Circle plot summarizing the number and strength of predicted ligand-receptor interactions between the eight major cell populations identified by scRNA-seq. **(B)** Heatmap showing the total number of ligand-receptor interactions for eight major cell populations. **(C)** Dot plot displaying the relative contribution of selected signaling pathways to the overall communication patterns, with the MDK, PTN, and PPIA pathways showing the higher enrichment in the tumor microenvironment. **(D)** Circle plot illustrating the expression distribution of representative ligands and receptors across all major cell populations. **(E–G)** Dot plot showing the scaled expression levels of ligands and receptors for MDK **(E)**, PTN **(F)**, and PPIA **(G)** signaling pathways across different cell types.

To further investigate how the transcriptional heterogeneity of SF-1^+^ tumor cells influences their crosstalk with the TME, we systematically inferred intercellular communication networks using CellChat. By integrating the ligand-receptor interactions with our single-cell transcriptomic profiles, we mapped the global communication architecture among the eight major cell populations (Figure 2A). Quantification of interaction numbers revealed that SF-1^+^ tumor cells, endothelial, macrophages, and fibroblasts constituted the dominant signaling hubs, exhibiting the highest number of outgoing and incoming interactions within the TME (Figure 2B). We next explored the specific signaling pathways mediating these interactions. Notably, the Midkine (MDK), Peptidylprolyl Isomerase A (PPIA) and Pleiotrophin (PTN) signaling families emerged as the most prominent communication channels between SF-1^+^ tumor cells and other stromal components (Figures 2C,D). Dot plot visualization of relevant ligand and receptor expression confirmed that the receptors for MDK and PTN, including SDC2, SDC4, NCL, and ALK, were broadly expressed on SF-1^+^ tumor cells, fibroblasts, and epithelial cells. Similarly, the ligands MDK and PTN were predominantly expressed by SF-1^+^ tumor cells and Epithelial (Figures 2E,F). Additionally, dot plots illustrated the expression patterns of PPIA and its receptor BSG. BSG was prominently expressed in SF-1^+^ tumor cells and epithelial cells, while PPIA was widely detected across the other seven cell types with the exception of neutrophils (Figure 2G). These findings indicate that SF-1^+^ tumor cells actively engage in pro-tumorigenic crosstalk via MDK and PTN signaling, potentially promoting angiogenesis, fibroblast activation, and an immunosuppressive milieu (Liran et al., 2020).

### 3.2 Characterization of SF-1^+^ tumor subclusters reveals distinct functional states

**FIGURE 3:**
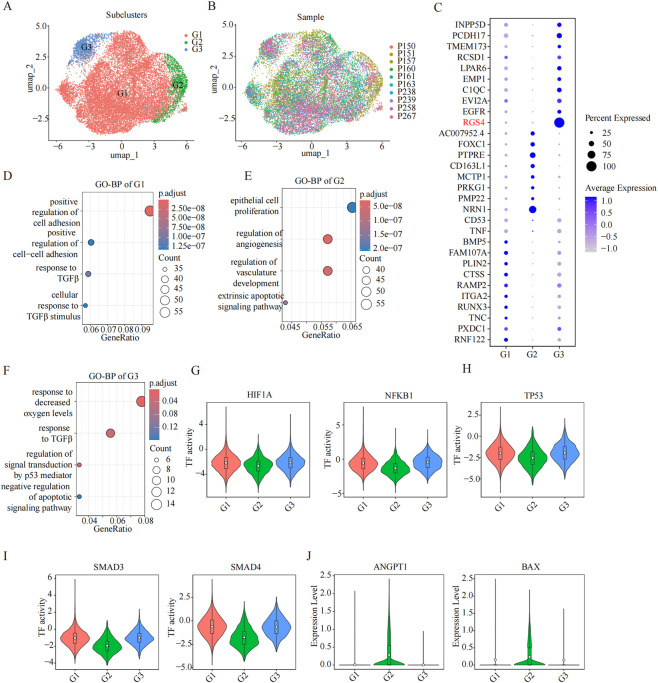
Characterization of SF-1^+^ tumor subclusters. **(A)** UMAP visualization showing three transcriptionally distinct subclusters in the SF-1^+^ tumor cell compartment (G1–G3). **(B)** UMAP representation of the 10 SF-1 lineage PitNETs samples mapped onto the SF-1 tumor subclusters. **(C)** Dot plot displaying the expression profiles of representative marker genes in the three SF-1 tumor subclusters. **(D–F)** The GO-BP enrichment analyses for differentially expressed genes in subclusters G1, G2, and G3. **(G–I)** Violin plots illustrating transcription factor activity scores for key signaling pathways across the three subclusters, including Hypoxia (HIF1A, NFKB1), p53 (TP53), and TGF-β (SMAD3, SMAD4). **(J)** Violin plots comparing the expression of genes associated with angiogenesis (ANGPT1) and apoptosis (BAX) across G1-G3.

Building on the tumor cell dominant percentage observed within the overall TME, we next focused our analysis on the predominant SF-1^+^ tumor cell compartment. Subclustering of these cells identified three transcriptionally distinct subgroups, designated G1, G2, and G3 (Figure 3A). All three subclusters were detected across the 10 patient samples, although their relative abundances varied, suggesting conserved yet heterogeneous biological states (Figure 3B). Differential expression analysis revealed unique marker gene signatures for each subgroup (Figure 3C). To explore their functional divergence, we performed GO Biological Process enrichment on the subgroup-specific signature genes, which uncovered distinctly enriched biological themes. We next interrogated the regulatory landscape by assessing transcription factor (TF) and pathway activity. Violin plot analyses showed that the hypoxia response pathway was most strongly activated in the G2 subgroup, whereas p53 signaling exhibited the highest activity in G3. In contrast, TGF-β signaling was increased primarily in G1 and G3 (Figures 3D–F). These pathway-level differences were consistent with the expression patterns of key functional markers and TFs. Specifically, activity of HIF1A and NF-κB, which are TFs associated with hypoxic stress along with Trp53, was elevated in G3 (Figures 3G,H). In contrast, SMAD3/4 activity, reflecting downstream TGF-β signaling, was enhanced in G1 and G3 (Figure 3I). Additionally, the pro-angiogenic factor ANGPT1 and the pro-apoptotic gene BAX were most highly expressed in the G2 subgroup (Figure 3J). Collectively, these analyses define three stable SF-1^+^ tumor cell subgroups with G1, G2, and G3, each exhibiting distinct transcriptional identities, specialized functional programs, and divergent regulatory network activities, underscoring the substantial intratumoral heterogeneity within SF-1 lineage PitNETs.

### 3.3 Integration of single-cell and bulk transcriptomic analysis reveals an *RGS4*-High subgroup

**FIGURE 4:**
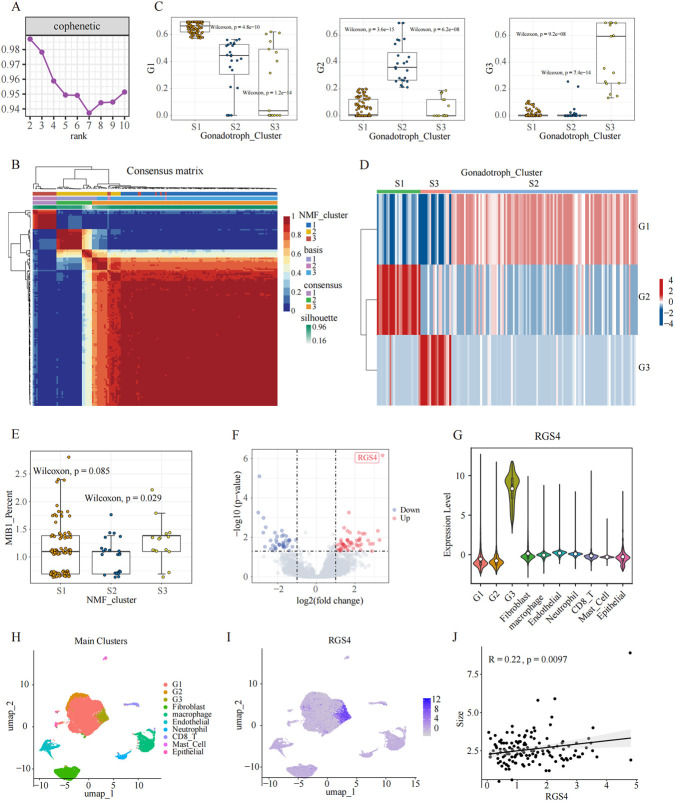
Integrated analysis identifies an *RGS4*-high subgroup. **(A)** Identification of three molecular subgroups from bulk RNA-seq data (n = 138 gonadotroph tumors) using NMF. The cophenetic curve when rank k was set as 2 to 10. **(B)** Consensus heatmap illustrating the stability of NMF clustering at k = 3, defining three distinct transcriptomic subgroups. **(C,D)** Relactive Abundance of single-cell-derived SF-1 tumor subclusters (G1-G3) across the three NMF-defined molecular subgroups, inferred using CIBERSORT-based deconvolution of bulk RNA-seq. **(E)** Boxplot comparing MIB-1 proliferation index across the three transcriptomic subgroups. **(F)** Volcano map displaying differentially expressed genes between the *RGS4*-high subgroup and other subgroups within the bulk RNA-seq dataset. **(G–I)** UMAP and violin plots showing *RGS4* expression patterns across SF-1 tumor cells in the scRNA-seq dataset. **(J)** Pearson correlation analysis showing a significant positive correlation between *RGS4* expression and tumor size.

To link the single-cell–defined tumor cell states with clinical phenotypes and identify potential drivers of the proliferative program, we integrated our single-cell dataset with an independent bulk RNA-seq cohort comprising 138 SF-1 lineage (Gonadotroph) PitNETs. Using CIBERSORT, we deconvoluted the bulk transcriptomes based on the single-cell–derived marker genes for the G1, G2, and G3 subgroups, thereby estimating the relative abundance of each cellular state in each bulk sample. These inferred cellular proportions were subsequently used as the input matrix for NMF clustering of the bulk cohort. NMF analysis consistently resolved three distinct molecular subgroups, designated S1–S3 (Figures 4A,B). Boxplots and heatmaps demonstrated clear differences in the distribution of G1, G2, and G3 signatures across these molecular subgroups, highlighting their divergent compositional architectures (Figures 4C,D). Importantly, the transcriptomic signatures of the G1–G3 subclusters were significantly enriched in S1-S3, respectively, supporting the robustness of the integrative classification. Clinical correlation analysis revealed that S3 characterized by strong enrichment of the G3 signature displayed a significantly higher MIB-1 proliferation index than the other subgroups (Figure 4E). This finding indicates that the G3-associated molecular subgroup corresponds to the most proliferative clinical phenotype.

To identify candidate drivers of the proliferative phenotype, we performed differential expression analysis comparing the S3 subgroup with all other tumors in the bulk RNA-seq cohort. *RGS4* emerged as the most significantly upregulated gene in this comparison, immediately drawing our attention (Figure 4F). Consistent with this finding, examination of the single-cell dataset confirmed that *RGS4* expression was markedly enriched within the G3 subgroup (Figures 4G–I), in agreement with the G3 marker profile identified in Figure 3C. Moreover, clinical association analysis revealed a significant positive correlation between *RGS4* expression and tumor size in the bulk cohort (Figure 4J), further implicating *RGS4* in tumor progression. Collectively, these findings identify *RGS4* as a defining feature of the G3 proliferative state and highlight its potential role as a key molecular driver of tumor growth in SF-1 lineage PitNETs.

## 3.4 *Rgs4* enhances cell proliferation in PitNETs cell lines *in vitro* and *in vivo*

**FIGURE 5:**
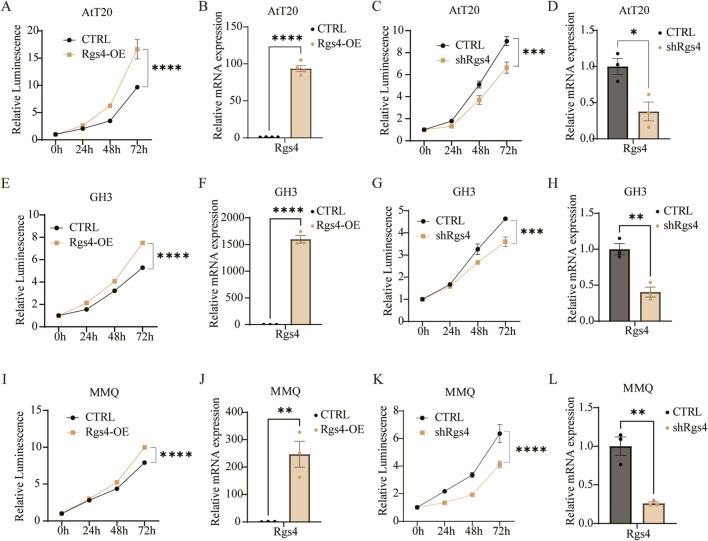
*Rgs4* promotes tumor proliferation *in vitro*. **(A,E,I)** CellTiter-Glo assessing cell viability following ***Rgs4*** overexpression in AtT20 cells, GH3 cells **(E)**, and MMQ cells **(I)**. **(B,F,J)** RT–qPCR analysis confirming ***Rgs4*** overexpression and quantifying downstream mRNA changes in AtT20 cells, GH3 cells **(F)**, and MMQ cells **(J)**. **(C,G,K)** CellTiter-Glo assays evaluating cell viability after ***Rgs4*** knockdown in AtT20 cells, GH3 cells **(G)**, and MMQ cells **(K)**. **(D,H,L)** RT-qPCR validation of ***Rgs4*** knockdown and associated transcriptional changes in AtT20 cells, GH3 cells **(H)**, and MMQ cells (**L**).

Given the strong association between *RGS4* expression and the proliferative G3/S3 subgroup identified in our transcriptomic analyses, we next sought to functionally validate its role in tumor cell biology. Stable *Rgs4*-overexpressing lines were generated in three PitNETs cell models, and successful overexpression was confirmed at the mRNA level by RT-qPCR (Figures 5B,F,J). *Rgs4* overexpression markedly enhanced cell proliferation in all three lines, as quantified by the CellTiter-Glo assay (Figures 5A,E,I). Conversely, lentiviral-mediated knockdown of *Rgs4*, as verified by RT-qPCR (Figures 5D,H,L), resulted in a significant reduction in proliferative capacity across all cell models (Figures 5C,G,K).

**FIGURE 6:**
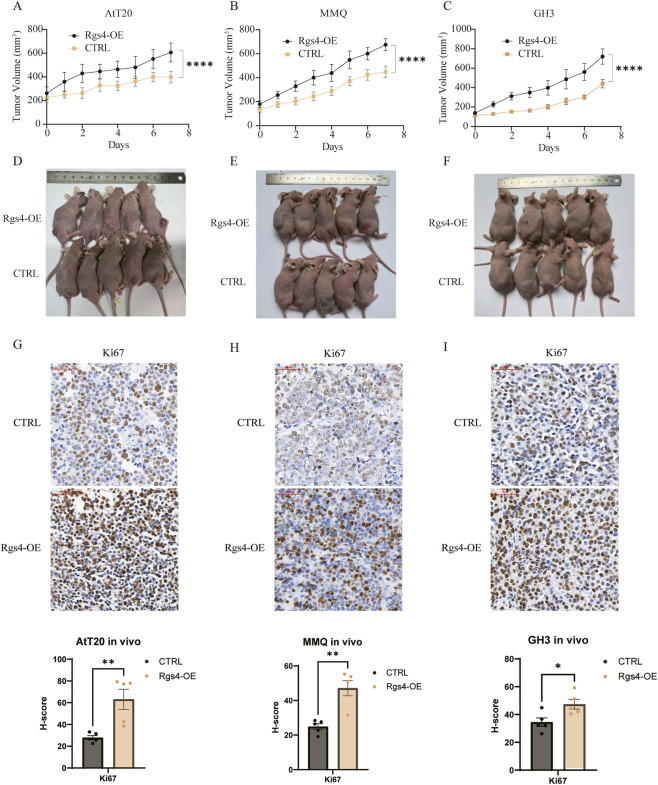
*Rgs4* promotes tumor proliferation *in vivo*. (**A–C)** Tumor growth curves from subcutaneous xenograft models generated by injecting AtT20 cells **(A)**, MMQ cells **(B)**, and GH3 cells **(C)**. (**D–F)** Representative images of subcutaneous xenograft tumors formed after injection of AtT20 cells **(D)**, with corresponding images for MMQ **(E)** and GH3 **(F)** cell–derived tumors. (**G–I)** Ki-67 expression and its H-score was detected and calculated by immunohistochemistry in AtT20 cells **(G)**, MMQ cells **(H)**, and GH3 cells **(I)**. P-value was calculated by student t test.

To further assess the oncogenic potential of *Rgs4 in vivo*, BALB/c nude mice were injected subcutaneously with the *Rgs4*-overexpressing stable lines. Tumor growth monitoring revealed that *Rgs4* overexpression significantly accelerated tumor progression in all three xenograft models (Figures 6A–F). Immunohistochemical analysis of Ki-67 expression in the xenograft tumors further confirmed that Rgs4 overexpression significantly enhanced proliferation, as evidenced by increased Ki-67 positivity across all three cell lines (Figures 6G–I). Together, these results indicate that *Rgs4* functions as a key promoter of PitNETs cell proliferation and tumor growth, reinforcing its role as a potential oncogenic driver in SF-1 lineage PitNETs.

### 3.5 Inhibition of RGS4 induces PitNETs cell lines apoptosis

**FIGURE 7:**
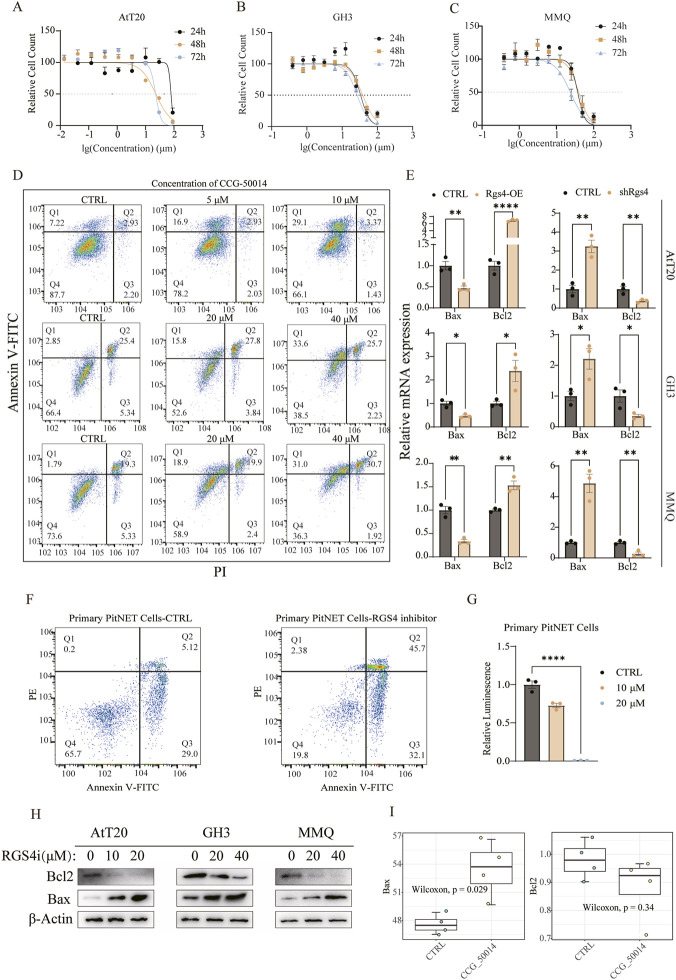
RGS4 inhibition induces apoptosis in PitNETs cells. **(A–C)** Cell viability of three pituitary tumor cell lines following treatment with the RGS4 inhibitor at multiple concentrations and time points, measured using the CellTiter-Glo luminescence assay. **(D)** Flow cytometry analysis assessing apoptosis rates in the three cell lines after exposure to different doses of the RGS4 inhibitor. **(E)** RT-qPCR quantification of Bax and Bcl-2 mRNA levels in the three cell lines following ***Rgs4*** overexpression or knockdown. **(F)** Apoptosis of primary PitNET cells following RGS4 inhibitor treatment was assessed by flow cytometry. **(G)** Cell viability of primary PitNET cells following RGS4 inhibitor treatment was measured using the CellTiter-Glo assay. **(H)** Western blot analysis evaluating protein expression of Bax and Bcl-2 in response to increasing concentrations of the RGS4 inhibitor in the three cell lines. **(I)** Transcriptomic profiling of AtT20 cells showing Bax and Bcl-2 expression changes after RGS4 inhibitor treatment (5 μM, 24 h).

Having demonstrated that RGS4 enhances tumor cell viability, we next evaluated its therapeutic potential and underlying mechanisms. Treatment of PitNETs cell lines with the RGS4 inhibitor CCG-50014 resulted in a robust suppression of cellular proliferation across all three models (Figures 7A–C). To determine whether this growth inhibition was associated with apoptosis, we conducted flow cytometry analysis following inhibitor treatment. In all cell lines, RGS4 inhibition significantly increased the proportion of apoptotic cells in a dose-dependent manner (Figure 7D), indicating that RGS4 supports tumor cell survival by restraining apoptotic pathways. We further assessed the expression of apoptosis-related regulators. *Rgs4* overexpression markedly decreased Bax mRNA levels while upregulating Bcl2, whereas *Rgs4* knockdown produced the opposite effect (Figure 7E). To further validate these findings in human-derived specimens, we performed additional experiments using primary human pituitary tumor cells. Apoptosis assays demonstrated that RGS4 inhibitor treatment significantly increased apoptosis (Figure 7F), while cell viability assays confirmed a dose-dependent suppression of proliferation in primary human pituitary tumor cells (Figure 7G). Correspondingly, RGS4 inhibition induced a progressive increase in Bax protein and a reduction in Bcl2 protein across all three cell lines (Figure 7H). To more comprehensively investigate the mechanism, transcriptome sequencing of AtT20 cells treated with the RGS4 inhibitor was performed. Differential expression analysis confirmed a significant upregulation of Bax and downregulation of Bcl2 at the transcript level (Figure 7I), consistent with the RT-qPCR and protein-level findings. These findings suggest that *Rgs4* promotes pituitary tumor cell survival by suppressing apoptosis through modulation of Bax and Bcl2, and that pharmacologic inhibition of RGS4 represents a promising therapeutic strategy.

## 3.6 RGS4 mediates its oncogenic effects by regulating p53 protein stability via the ubiquitin–proteasome system

**FIGURE 8:**
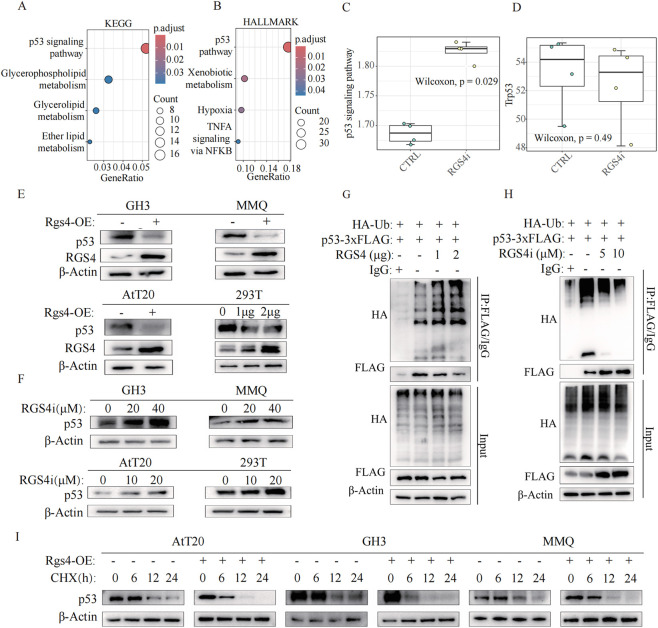
RGS4 regulates p53 protein ubiquitination and stability. **(A,B)** KEGG pathway and HALLMARK gene set enrichment analyses of transcriptomic data from AtT20 cells treated with an RGS4 inhibitor. **(C)** ssGSEA-based quantification of p53 signaling pathway activity in AtT20 transcriptome datasets following RGS4 inhibition. **(D)** Trp53 mRNA expression from RNA-seq data in RGS4 inhibitor–treated AtT20 cells. **(E)** Western blot analysis of p53 and RGS4 protein levels after ***Rgs4*** overexpression in four cell lines. **(F)** Western blot analysis of p53 and RGS4 expression in response to 24 h of exposure to increasing concentrations of the RGS4 inhibitor in four cell lines. **(G)** IP assays showing increased p53 ubiquitination in 293T cells co-transfected with HA-Ub, p53-3×FLAG, and *RGS4* expression plasmid. **(H)** Co-IP assays demonstrating altered p53 ubiquitination in 293T cells co-transfected with HA-Ub and p53-3×FLAG following treatment with an RGS4 inhibitor. **(I)** CHX chase assays showing p53 protein stability in *RGS4*-overexpressing cell lines at the indicated time points.

To elucidate the upstream regulatory mechanisms associated with RGS4 inhibition induced apoptosis, we conducted functional enrichment analysis on bulk RNA-seq of AtT20. KEGG and HALLMARK enrichment analyses of the differentially expressed genes revealed a marked activation of the p53 signaling pathway following RGS4 inhibition (Figures 8A,B). Consistently, ssGSEA-based pathway activity scoring demonstrated a significant elevation in p53 pathway activity upon inhibitor treatment (Figure 8C), suggesting that RGS4 may promote tumor cell proliferation through modulation of p53 signaling. To further explore the relationship between RGS4 and p53, we examined p53 protein levels under conditions of altered RGS4 expression. Western blot analysis showed that RGS4 overexpression substantially reduced p53 protein abundance across multiple pituitary adenoma cell lines (Figure 8E), whereas pharmacologic inhibition of RGS4 led to a dose-dependent upregulation of p53 protein (Figure 8F). Notably, transcriptomic analysis of inhibitor-treated AtT20 cells revealed no significant change in Trp53 mRNA levels (Figure 8D), despite the pronounced alterations observed at the protein level. This discrepancy indicates that RGS4 regulates p53 not at the transcriptional level but likely through post-translational mechanisms.

According to previous studies, ubiquitin-mediated degradation was an important post-transcriptional regulation mechanism of p53 (Wei et al., 2024; Zhang et al., 2019; Hou et al., 2024). Based on this, we hypothesized that RGS4 may similarly modulate p53 stability through ubiquitin-mediated degradation. Immunoprecipitation assays provided direct support for this mechanism: RGS4 overexpression markedly increased p53 ubiquitination (Figure 8G), whereas pharmacologic inhibition of RGS4 substantially reduced ubiquitinated p53 levels (Figure 8H). These findings indicate that RGS4 promotes tumorigenesis by facilitating p53 ubiquitination. To further validate that RGS4 influences p53 stability, we performed cycloheximide (CHX) assays to block new protein synthesis. Western blot analysis demonstrated that RGS4 overexpression accelerated the time-dependent degradation of p53 in the presence of CHX (Figure 8I), confirming its role in enhancing p53 degradation. Collectively, these results define a previously unrecognized mechanism whereby RGS4 promotes pituitary tumor cell proliferation by destabilizing the tumor suppressor p53. RGS4 enhances p53 ubiquitination, thereby driving its accelerated proteasomal degradation.

## 4 Discussion

In this integrated investigation, we delineate a previously unrecognized molecular pathway that underlies the proliferative behavior of SF-1 lineage PitNETs. Through the systematic integration of single-cell transcriptomic profiling with bulk RNA-seq data from a large independent patient cohort, we identified a distinct SF-1^+^ PitNETs subtype defined by markedly elevated *RGS4* expression. This convergence of single-cell and population-level data provided compelling evidence that *RGS4* marks a biologically aggressive cellular state within SF-1 lineage tumors. Functional perturbation experiments across multiple *in vitro* and *in vivo* models further established *Rgs4* as a critical regulator of PitNETs biology. Overexpression of *Rgs4* markedly enhanced tumor cell proliferation and tumor growth, whereas its inhibition or knockdown significantly impaired viability and induced apoptosis. These complementary approaches confirm that *RGS4* is not merely a correlational biomarker but a functional driver of tumor progression. Mechanistically, we uncovered that *RGS4* exerts its oncogenic activity by modulating the stability of the tumor suppressor p53. RGS4 promotes p53 ubiquitination and accelerates its proteasomal degradation, thereby dampening p53-mediated apoptotic and anti-proliferative signaling. This mechanistic insight connects *RGS4* activity directly to a canonical tumor suppressor pathway and provides a coherent explanation for the proliferative phenotype observed in the *RGS4*-high subgroup. Collectively, these findings position RGS4 as a central intracellular driver of tumor proliferation in SF-1 lineage PitNETs and highlight it as a promising therapeutic target. Targeting RGS4 or its downstream signaling axis may offer a novel strategy for treating this clinically challenging tumor subtype.

PitNETs are among the most common intracranial neoplasms and display substantial clinical and biological heterogeneity. These tumors frequently impair endocrine function and produce mass-related neurological symptoms, leading to significant morbidity. Although classified as benign, PitNETs exhibit marked variability in proliferative capacity, invasiveness, and recurrence risk, posing considerable challenges in their clinical management (Dottermusch, 2025; Han et al., 2021; Melmed et al., 2022). Standard therapeutic approaches, including surgery, radiotherapy, and medical therapy, often achieve incomplete disease control or are associated with limited tolerability, underscoring the need for a deeper understanding of their molecular drivers to facilitate development of more effective treatment strategies (Saeger and Koch, 2021; Toade et al., 2023). Our single-cell transcriptomic profiling of SF-1 lineage PitNETs revealed a complex tumor microenvironment composed of eight major cellular populations, with SF-1^+^ tumor cells forming the predominant compartment. Importantly, detailed subcluster analysis of the SF-1^+^ tumor cells uncovered substantial transcriptional heterogeneity. Through subcluster analysis, we identified a highly proliferative S3 subtype characterized by markedly elevated *RGS4* expression in SF1 lineage PitNETs. RGS4, a member of the regulator of G-protein signaling family, is a key modulator of GPCR-mediated signaling pathways that govern cell proliferation, differentiation, and survival (Tan et al., 2025). Its elevated expression in aggressive tumor cells suggests a selective advantage in maintaining oncogenic characteristics, potentially by attenuating inhibitory G-protein pathways (Lyu et al., 2020). The strong enrichment of *RGS4* in the S3 subcluster implicates it as a functional driver of this proliferative phenotype, a concept supported by its established pro-tumorigenic roles in other cancer contexts (Guillot et al., 2021). Within PitNETs, however, the mechanistic contribution of *RGS4* has not been previously defined. The positive correlation between *RGS4* expression and tumor size observed in our cohort further supports its involvement in promoting tumor expansion, potentially through regulation of intrinsic growth and survival pathways. Together, our findings identify the *RGS4*-high subgroup and establish *RGS4* as a biologically meaningful determinant of the proliferative landscape in SF-1 lineage PitNETs.

Functional assays confirmed that RGS4 exerts a direct regulatory influence on PitNETs cell fate: its overexpression markedly enhanced the proliferation of PitNET-derived cell lines, whereas its suppression induced pronounced apoptosis. These findings underscore the oncogenic potential of *RGS4* and parallel observations in osteosarcoma, where *RGS4* promotes tumor growth and metastasis through interactions with transcriptional regulators such as TWIST1 and by modulating epithelial–mesenchymal transition-associated pathways (Tan et al., 2025; Chen et al., 2025). The pro-proliferative effects of *RGS4* in PitNETs appear to involve attenuation of the p53 signaling axis. We demonstrated that RGS4 modulates p53 primarily at the post-translational level, as RGS4 overexpression reduced p53 protein abundance, whereas its inhibition resulted in increased p53 protein level despite no detectable changes in Trp53 mRNA expression. Therefore, by downregulating p53 protein levels, RGS4 may enable tumor cells to evade p53-mediated cell cycle checkpoints and apoptosis, thereby sustaining uncontrolled proliferation. Therapeutically targeting this RGS4-p53 axis could restore p53 functionality and reinstate tumor suppressive mechanisms, offering a promising and biologically rational avenue for intervention in SF-1 lineage PitNETs.

## 5 Conclusion

This study revealed a highly proliferative tumor subgroup defined by elevated expression of RGS4. We demonstrate that RGS4 drives tumor proliferation by promoting ubiquitin-mediated degradation of p53. These findings establish the RGS4-p53 axis as a key mechanistic pathway and a promising therapeutic target for this disease.

## Data Availability

The datasets presented in this study can be found in online repositories. The names of the repository/repositories and accession number(s) can be found in the article/Supplementary Material.
