# Supplementary figures and images for: Integrating single-cell and bulk RNA sequencing data reveals RGS4 as a functional driver in a proliferative subgroup of SF-1 lineage PitNETs
Source: Front Cell Dev Biol. 2026 Apr 22;14:1815682. doi: 10.3389/fcell.2026.1815682 (PMC13144082; doi:10.3389/fcell.2026.1815682)

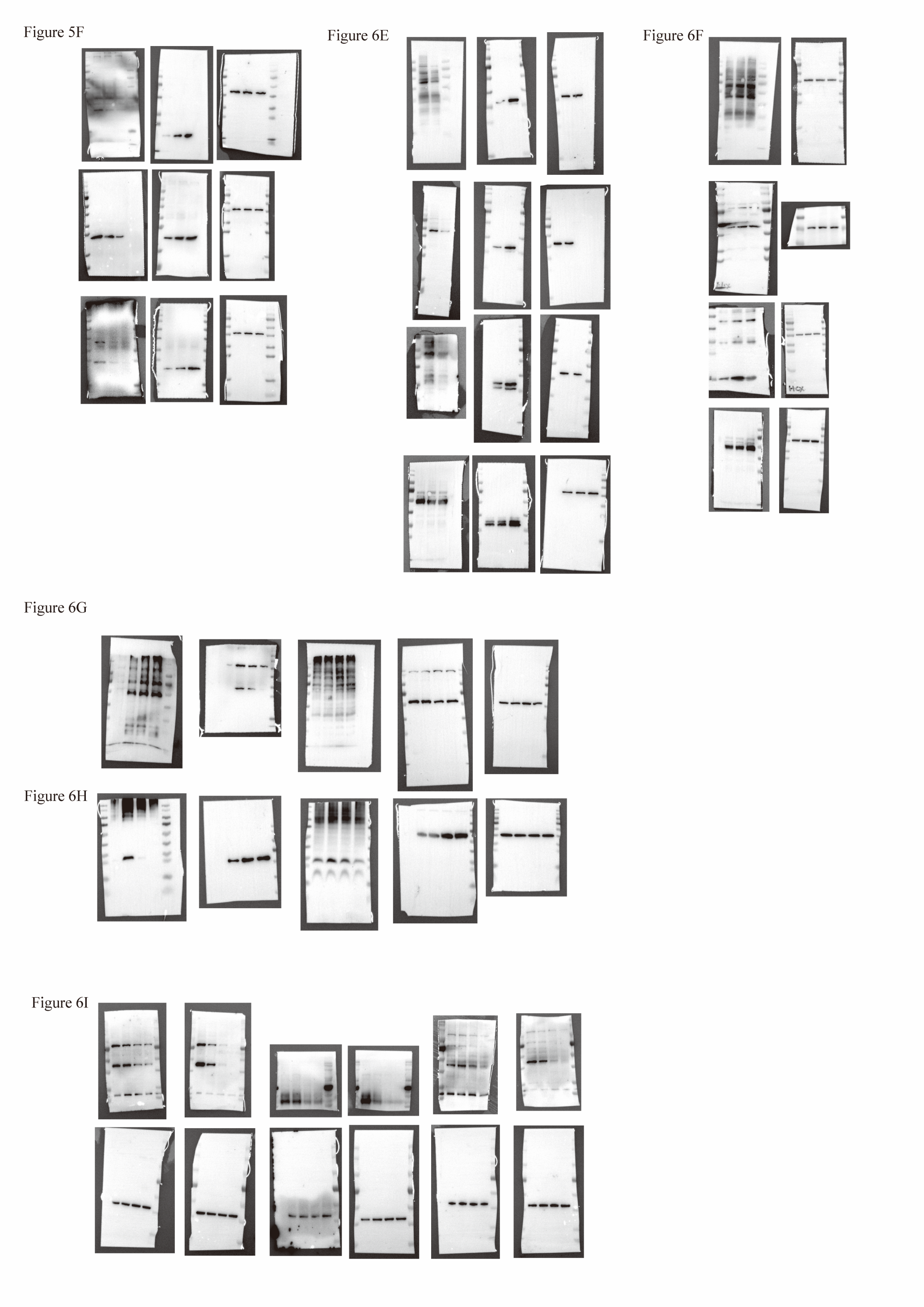

Supplement: Supplementary file 2 [file Image1.tiff]
